# Expanding the Genetic Spectrum in *IMPG1* and *IMPG2* Retinopathy

**DOI:** 10.3390/genes16121474

**Published:** 2025-12-09

**Authors:** Saoud Al-Khuzaei, Ahmed K. Shalaby, Jing Yu, Morag Shanks, Penny Clouston, Robert E. MacLaren, Stephanie Halford, Samantha R. De Silva, Susan M. Downes

**Affiliations:** 1 Oxford Eye Hospital, John Radcliffe Hospital, Oxford University Hospitals-NHS Foundation Trust, Oxford OX3 9DU, UK; 2 Nuffield Laboratory of Ophthalmology, Nuffield Department of Clinical Neuroscience, University of Oxford, John Radcliffe Hospital, Headley Way, Oxford OX3 9DU, UK; 3 Oxford Medical Genetics Laboratories, Oxford University Hospitals-NHS Foundation Trust, Oxford OX3 7LE, UK

**Keywords:** *IMPG1*, *IMPG2*, retinitis pigmentosa, adult vitelliform macular dystrophy

## Abstract

***Background:*** Pathogenic variants in interphotoreceptor matrix proteoglycan 1 (*IMPG1*) have been associated with autosomal dominant and recessive retinitis pigmentosa (RP) and autosomal dominant adult vitelliform macular dystrophy (AVMD). Monoallelic pathogenic variants in *IMPG2* have been linked to maculopathy and biallelic variants to RP with early onset macular atrophy. Herein we characterise the phenotypic and genotypic features of patients with IMPG1/IMPG2 retinopathy and report novel variants. ***Methods***: Patients with *IMPG1* and *IMPG2* variants and compatible phenotypes were retrospectively identified. Clinical data were obtained from reviewing the medical records. Phenotypic data included visual acuity, imaging included ultra-widefield pseudo-colour, fundus autofluorescence, and optical coherence tomography (OCT). Genetic testing was performed using next generation sequencing (NGS). Variant pathogenicity was investigated using in silico analysis (SIFT, PolyPhen-2, mutation taster, SpliceAI). The evolutionary conservation of novel missense variants was also investigated. ***Results***: A total of 13 unrelated patients were identified: 2 (1 male; 1 female) with *IMPG1* retinopathy and 11 (7 male; 4 female) with *IMPG2* retinopathy. Both *IMPG1* retinopathy patients were monoallelic: one patient had adult vitelliform macular dystrophy (AVMD) with drusenoid changes while the other had pattern dystrophy (PD), and they presented to clinic at age 81 and 72 years, respectively. There were 5 monoallelic *IMPG2* retinopathy patients with a maculopathy phenotype, of whom 1 had PD and 4 had AVMD. The mean age of symptom onset of this group was 54.2 ± 11.8 years, mean age at presentation was 54.8 ± 11.5 years, and mean BCVAs were 0.15 ± 0.12 logMAR OD and −0.01 ± 0.12 logMAR OS. Six biallelic *IMPG2* patients had RP with maculopathy, where the mean age of onset symptom onset was 18.4 years, mean age at examination was 68.7 years, and mean BCVAs were 1.90 logMAR OD and 1.82 logMAR OS. Variants in *IMPG1* included one missense and one exon deletion. A total of 11 different *IMPG2* variants were identified (4 missense, 7 truncating). A splicing defect was predicted for the c.871C>A p.(Arg291Ser) missense *IMPG2* variant. One *IMPG1* and five *IMPG2* variants were novel. ***Conclusions***: This study describes the phenotypic spectrum of *IMPG1*/*IMPG2* retinopathy and six novel variants are reported. The phenotypes of PD and AVMD in monoallelic *IMPG2* patients may result from haploinsufficiency, supported by the presence of truncating variants in both monoallelic and biallelic cases. The identification of novel variants expands the known genetic landscape of *IMPG1* and *IMPG2* retinopathies. These findings contribute to diagnostic accuracy, informed patient counselling regarding inheritance pattern, and may help guide recruitment for future therapeutic interventions.

## 1. Introduction

The term inherited retinal diseases (IRDs) is used to describe a group of genetically determined retinal conditions, including retinitis pigmentosa (RP) and macular dystrophies [1]. IRDs collectively represent a leading cause of blindness in the working age population in England and Wales [2,3]. To date, pathogenic variants in over 330 genes have been associated with IRDs (https://retnet.org/, accessed 7 January 2025), emphasising the genetic heterogeneity of these conditions [4].

Pathogenic variants in interphotoreceptor matrix proteoglycan 1 (*IMPG1*) and two (*IMPG2*) genes have been reported in association with a spectrum of retinal phenotypes [5,6,7,8]. *IMPG1*, located at 6q14.1, encodes a 797 amino acid glycoprotein previously termed sialoprotein associated with cones and rods (SPACR) [9,10]. *IMPG2,* located at 3q12.3, encodes a 1241 amino acid protein that was previously referred to as sialoprotein associated with cones and rods proteoglycans (SPACRCAN), which is structurally similar to SPACR [11,12,13].

Both genes encode glycoproteins that are integral to the interphotoreceptor matrix (IPM) [14]. The IPM is a carbohydrate-rich extracellular matrix that extends from the Muller cells and the retinal pigment epithelium (RPE) and surrounds the inner and outer segments of the photoreceptors [15,16,17]. The IPM has been shown to play an important role in photoreceptor homeostasis, nutrient and metabolite transport, retinoid transport, intercellular communication, photoreceptor differentiation and maintenance, retinal attachment, photoreceptor alignment, and interaction with outer segment phagocytosis [17,18,19].

IMPG1 and IMPG2 are expressed in rod and cone photoreceptors [10,13,20] where they bind to chondroitin sulphate and hyaluronan and thus contribute to the stabilisation of the IPM scaffold [12,21,22]. Both proteins are densely glycosylated, chondroitin sulphate-rich (Chs), and contain two SEA (sperm protein, enterokinase, and agrin) domains [12,13].

**Figure 1:**
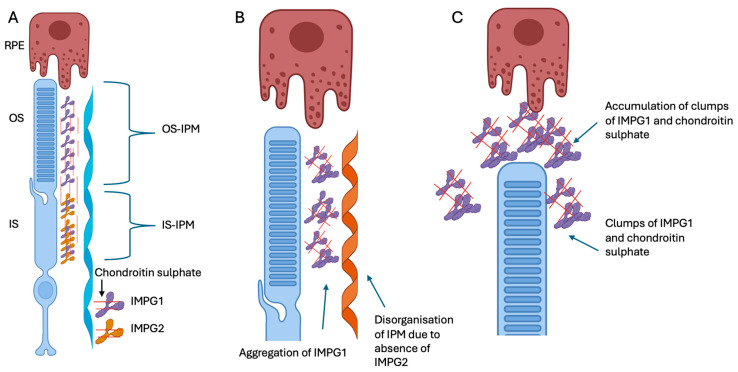
Schematic representation of IMPG1 and IMPG2 along with the photoreceptor and retinal pigment epithelium cell (RPE). (**A**) Schematic representation showing IMPG1 (purple) anchored to the photoreceptor outer and inner segments (OS and IS) and IMPG2 (yellow) anchored to the photoreceptor IS facing the IPM. The IMPG2 interacts with the IPM, thus allowing the secreted IMPG1 to follow an organised conformation. The IMPG1 then follows the OS as they mature and are phagocytosed by the retinal pigment epithelium (RPE) cells. (**B**) Absence of IMPG2 stops the organised conformation of the secreted IMPG1. (**C**) The disorganised IMPG1 forms clumps with chondroitin sulphate which affect the clearance of IPM by the RPE, and this material is thought to accumulate within the subretinal space and eventually results in the vitelliform lesion. Figure was adapted from Salido et al. [14] created in https://BioRender.com (accessed on 30 October 2025).

IMPG1 is distributed across both the inner and outer segments, whilst IMPG2 is localised to the inner segments. Despite the differences in localisation, Salido et al. found that the correct localisation of IMPG1 is dependent on IMPG2 (Figure 1) [14]. Moreover, their group recently also showed that the trafficking of the extracellular portion of IMPG2 from the IPM near the inner segment to the outer segment of the IPM is dependent on the presence of IMPG1, suggesting that the proteins were co-dependant [23]. This inter-dependence between the proteins could explain the overlapping and variable phenotypes that have been associated with *IMPG1* and *IMPG2* variants.

Pathogenic variants in *IMPG1* have been associated with both autosomal dominant and recessive RP and adult vitelliform macular dystrophy (AVMD) [6,8,24,25,26]. *IMPG2* variants are associated with childhood-onset autosomal recessive retinitis pigmentosa (arRP) with early maculopathy in biallelic cases and AVMD and PD in monoallelic cases [5,7,8,27]. Follow-up studies on *IMPG2*-related AVMD by Brandl et al., showed gradual resorption of the hyper-reflective material that is located above the preserved RPE/Bruch’s membrane complex, resulting in an empty dome-shaped optical cavity. In some cases, this cavity was subsequently refilled with material, followed by a collapse of the cavity and development of central macular atrophy with RPE loss [8].

Phenotypic variability has been described in *IMPG2* retinopathy, including incomplete penetrance [7,8] and mild foveal changes in asymptomatic carriers, such as mild foveal RPE thickening, interdigitation zone loss/fading, and the presence of faint hyper-reflective bands in the space between the foveal ellipsoid zone (EZ) and RPE [6,7,8,27,28]. Haploinsufficiency has been proposed as an underlying mechanism for maculopathy phenotypes in monoallelic *IMPG2* retinopathy cases [7].

The current literature on variability in the phenotype and appearance of *IMPG1* and *IMPG2* retinopathy remains limited with only small cohort studies having been reported to date. This study adds to the phenotypic characterisation of *IMPG1* and *IMPG2* retinopathies, describing phenotypic variability and exploring genotype–phenotype correlations hereby adding to the current knowledge base and thus aiding diagnosis.

## 2. Materials and Methods

### 2.1. Patient Recruitment

Patients carrying at least one variant in *IMPG1* or *IMPG2* were identified retrospectively from the Oxford University Hospitals Medical Genetics Laboratory database. Patients were included in the study if their phenotype was consistent with an *IMPG*-related retinopathy. Patients were excluded if they had variants in other IRD genes that could independently explain their phenotype. This study was conducted in accordance with the Declaration of Helsinki with Ethics approval obtained from the local research ethics committee (reference 08/H0302/96).

### 2.2. Clinical Data

All patients were reviewed in a specialist ophthalmic genetics clinic at the Oxford Eye Hospital, where a detailed medical history and clinical examination was performed. Clinical data included baseline age, age of onset, sex, family history, symptoms, and best corrected visual acuity (BCVA). The BCVA was recorded on Snellen and Logarithm of the Minimum Angle of Resolution (LogMAR) charts and all BCVAs were converted to logMAR for the purposes of statistical analysis. A BCVA of counting fingers (CF) was represented by 2.5 logMAR and 3.0 logMAR for hand movements [29]. Multi-modal imaging included Optos pseudocolour widefield imaging (Optomap A10022; Optos Ltd., Dunfermline, Scotland), short wavelength fundus autofluorescence imaging (FAF) (Spectralis; Heidelberg Engineering, Heidelberg, Germany), and optical coherence tomography (OCT) imaging (SD-OCT; Spectralis, Heidelberg Engineering, Heidelberg, Germany).

### 2.3. Molecular Genetics Analysis

Genetic testing was performed at the Oxford Regional Genetics Laboratory, Oxford University Hospitals NHS Foundation Trust, as part of the patient’s routine care. Sequencing was performed based on the appropriate method available at the Oxford Medical Genetic Laboratory at the time of patient presentation. Enrichment of the *IMPG1* and *IMPG2* genes was achieved using a customised HaloPlex enrichment system kit (Agilent Technologies, Santa Clara, CA, USA) that was designed to capture the coding exons and 10bp of flanking introns on the phenotype-led gene panel [30]. Next generation sequencing (NGS) was performed using an Illumina MiSeq instrument (Illumina, San Diego, CA, USA) machine using a MiSeq v3 kit as per the manufacturer’s instructions [30]. Detected variants were confirmed using Sanger sequencing.

Chromosome position was based on the GRCh37/hg19 build and nucleotide and protein numbering was based on transcript NM_001563 for *IMPG1* and NM_016247.4 for *IMPG2*. Protein alignment was performed using protein transcripts NP_001554.2 for IMPG1 and NP_057331.2 for IMPG2. The pathogenicity of variants in *IMPG1* and *IMPG2* was investigated using the following three different in silico analysis programmes: Mutation Taster (http://www.mutationtaster.org/, accessed 7 January 2025), PolyPhen2 (http://genetics.bwh.harvard.edu/pph2/, accessed 7 January 2025), and Sorting Intolerant from Tolerance (SIFT) (http://sift.jcvi.org/, accessed 7 January 2025). The combined annotation-dependant duplication (CADD) score for all variants was also calculated (https://cadd.gs.washington.edu, accessed 7 January 2025) [31]. Missense variants were also analysed using alphamisse [32]. Defects in splicing were investigated using SpliceAI (https://spliceailookup.broadinstitute.org, accessed 7 January 2025) [33]. The variant frequency was checked using the Exome Aggregation Consortium (ExAC) and GnomAD databases (https://gnomad.broadinstitute.org, accessed 7 January 2025). Variants with a minor allele frequency of >0.1% were excluded. The pathogenicity of all variants was classified according to the American College of Medical Genetics and Genomics (ACMG) criteria. Variants were considered novel if they were not reported in the LOVD (https://www.lovd.nl/, accessed 7 January 2025) or Clinvar (https://www.ncbi.nlm.nih.gov/clinvar/, accessed 7 January 2025) databases.

Evolutionary conservation of novel missense variants was investigated using Clustal Omega (https://www.ebi.ac.uk/jdispatcher/msa/clustalo, accessed 7 January 2025). Accession numbers for IMPG1 were Human (Homo sapiens) NP_001554.2; Chimpanzee (*Pan troglodytes*) XP_009449933.3; cow (*Bos taurus*) NP_776787.1; rat (Rattus norvegicus) BAJ33461.1; mouse (*Mus musculus*) AAH22970.1; chicken (*Gallus gallus*) XP_046794338.1; and frog (*Xenopus tropicalis*) XP_031758749.1. Accession numbers for IMPG2 were Human (*Homo sapiens*) NP_057331.2; Chimpanzee (*Pan troglodytes*) PNI64296.1; cow (*Bos taurus*) XP_027417236.1; rat (*Rattus norvegicus*) XP_032756196.1; mouse (*Mus musculus*) NP_777365.2; chicken (*Gallus gallus*) NP_001038104.3; and frog (*Xenopus tropicalis*) XP_012813076.2.

## 3. Results

### 3.1. Clinical Characteristics and Phenotype

This study included 13 patients with *IMPG* related retinopathy, including 2 *IMPG1* (1 male, 1 female) and 11 *IMPG2* cases (7 male, 4 female). The genotypic and phenotypic data for all 13 patients are summarised in Table 1 and Table 2 (additional phenotypic data are available in Supplementary Table S1).

**Figure 2:**
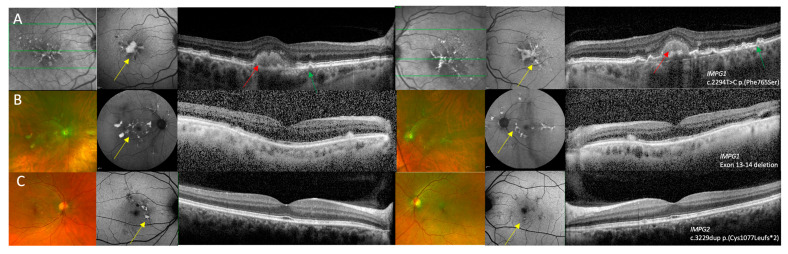
Multi-modal imaging of right and left eyes in *IMPG1* and *IMPG2* retinopathy patients with a pattern dystrophy phenotype. (**A**) Near infra-red reflectance image showing hyper-reflective macular lesions, fundus autofluorescence (FAF) imaging showing a foveal lesion with raised AF with adjacent fleck-like lesions, and optical coherence tomography (OCT) imaging showing vitelliform lesions with drusen-like changes in patient 1 who was heterozygous for the c.2294T>C p.(Phe765Ser) variant in *IMPG1*. (**B**) Optos ultra-widefield imaging showing multi-focal yellow deposits within the posterior pole, FAF showing raised AF signal associated with the multi-focal lesions, and OCT imaging showing subretinal hyper-reflective changes that correspond to the lesions seen on pseudocolour and FAF images in patient 2 who was heterozygous for a deletion in exons 13–14 of *IMPG1*. (**C**) Optos ultra-widefield imaging showing perifoveal fleck-like changes, FAF images show that some flecks had raised AF signal while other areas had reduced AF where there was RPE loss. The OCT images showed relatively preserved retinal architecture in the right eye and a small perifoveal hyper-reflective lesion in the left eye in patient 3 who was heterozygous for the c.3229dup p.(Cys1077Leufs*2) variant in *IMPG2*. Features suggestive of PD are shown by the yellow arrows, AVMD shown by the red arrows, and drusen-like changes are highlighted by the green arrows.

Both patients with *IMPG1* retinopathy were monoallelic and had a PD phenotype on fundus imaging. At baseline examination, patient 1 was aged 81 years and had a BCVA of 0.42 logMAR OD and 0.52 logMAR OS, and patient 2 was aged 72 years and had BCVA of −0.10 logMAR OD and −0.10 logMAR OS which deteriorated to 0.18 logMAR OU 14 years later. Fundus autofluorescence imaging showed foveal hyper-fluorescent lesions in patient 1 and dense hyper-fluorescent fleck-like changes in the posterior pole in patient 2. The OCT in patient 1 showed bilateral adult vitelliform lesions associated with a pigment epithelial detachment and drusenoid changes (Figure 2A,B). The choroid appeared thickened in bilaterally in patients 1 and 3.

There were five monoallelic patients with variants in *IMPG2* with maculopathy which included PD in 1 patient and AVMD in 4 patients. Mean age at baseline examination was 54.8 ± 11.5 years. Mean baseline BCVA were 0.15 ± 0.12 logMAR OD and −0.01 ± 0.12 logMAR OS. Follow-up data were available for three patients and these showed stable vision of 0.00 logMAR OU in patient 3 over 15 years, a deterioration from 0.30 logMAR OD and 0.00 logMAR OS to 0.90 logMAR OD and 0.60 logMAR OS in patient 4, and a deterioration from 0.26 logMAR OD and −0.14 logMAR OS to 0.40 logMAR OD and 0.80 logMAR OS over 8 years in patient 5. Four patients were asymptomatic at baseline examination and were referred by an optician when they were incidentally found to have macular changes during routine appointments. One patient (patient 4) reported central scotoma, delayed dark adaptation, and photosensitivity aged 61.

**Figure 3:**
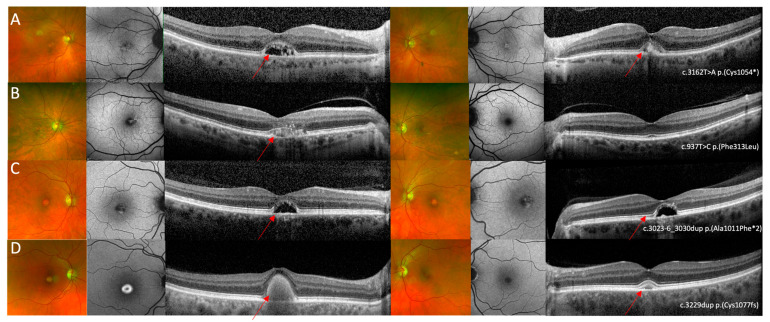
Multi-modal imaging in *IMPG2* retinopathy cases with an adult vitelliform maculopathy phenotype. (**A**) Optos ultrawide-field pseudo colour and fundus autofluorescence imaging shows mild bilateral foveal changes and optical coherence tomography shows bilateral vitelliform lesions with the left eye containing more hyper-reflective material compared to the right. Images relate to patient 4 who was heterozygous for the c.3162T>A p.(Cys1054*) variant in *IMPG2*. (**B**) Optos ultrawide-field pseudocolour imaging did not show specific retinal changes, FAF in the right eye shows a small region of raised AF in the right eye and was within normal limits in the left eye, and OCT imaging shows a shallow vitelliform lesion associated with hyper-reflective dots in the right eye and relatively preserved retinal architecture in the left eye. Images relate to patient 5 who was heterozygous for the c.937T>C p.(Phe313Leu) variant in *IMPG2*. (**C**) Optos ultrawide-field pseudo colour imaging showed bilateral foveal lesions, seen as bilateral patchy areas of raised signal on AF imaging; OCT imaging shows bilateral vitelliform lesions that did not contain hyper-reflective material. Images relate to patient 6 who was heterozygous for the c.3023-6_3030dup p.(Ala1011Phefs*2) variant in *IMPG2* (**D**) Optos ultrawide-field pseudo colour imaging shows a yellow foveal lesion in the right eye and no significant changes in the left eye, FAF shows a foveal lesion associated with raised AF signal in the right eye and no specific retinal changes in the left eye, and OCT imaging shows bilateral vitelliform lesions with the lesion in the right being larger compared to the left eye. Images relate to patient 7 who was heterozygous for the c.3229dup p.(Cys1077Leufs*2) variant in *IMPG2*. The red arrows highlight the AVMD lesions.

Patient 3 had a PD phenotype characterised by the presence of perifoveal fleck like changes on FAF and relative preservation of retinal architecture on OCT imaging with some EZ elevations in regions corresponding to the fleck-like changes (Figure 1C). In the four patients with AVMD, pseudocolour images showed a foveal yellow spot which was associated with variable amount of raised AF signal and OCT showed a foveal vitelliform lesion (Figure 3A–D). Patient 5 was the only patient with unilateral AVMD lesion that was also associated with hyper-reflective dots in the adjacent outer nuclear layer (Figure 3 C). The AVMD lesions were asymmetrical in patient 7 (Figure 3D). The choroid appeared to be thickened bilaterally in patients 6 and 7.

**Figure 4:**
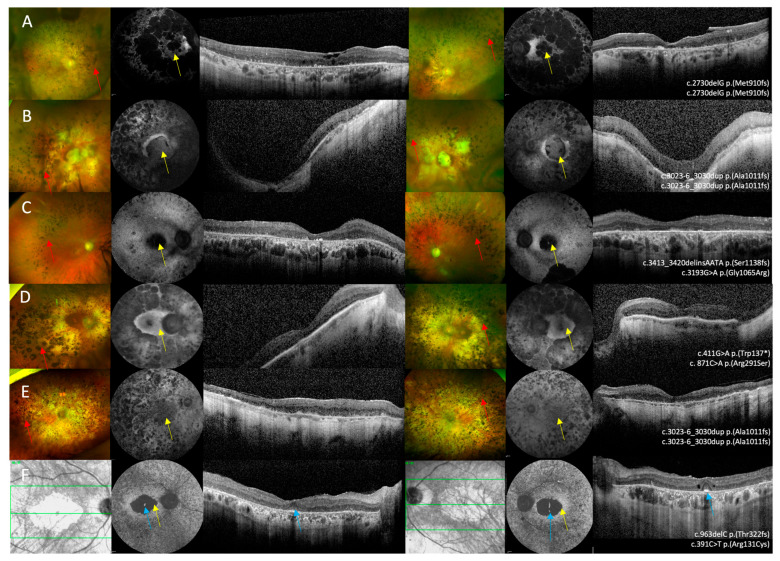
Multi-modal imaging in *IMPG2* retinopathy patients with retinitis pigmentosa and macular atrophy. (**A**–**E**) Optos ultrawide-field pseudo colour imaging showing macular atrophy and retinal atrophy in the mid-peripheral retina that was associated with pigmentary deposition, vascular attenuation, and optic disc pallor. (**F**) The near infra-red reflectance image in patient 13 shows bilateral macular atrophy. (**A**–**F**) Fundus autofluorescence imaging showing macular atrophy associated with varying degrees of retinal atrophy and mid-peripheral changes. (**A**–**F**) Optical coherence tomography imaging shows extensive outer retinal loss in all six patients. (**F**) OCT imaging in patient 13 shows bilateral foveal sparing disease. Bone spicule pigmentary deposition is shown by the red arrows, macular atrophy is highlighted by the yellow arrows, and the blue arrows shows the spared foveal tissue.

All six patients biallelic for *IMPG2* variants had an RP phenotype associated with maculopathy characterised by an atrophic macula. Mean age of symptom onset was 18.4 ± 16.0 years and mean age at baseline examination was 68.7 ± 12.3 years. Follow-up data were available for four patients; mean follow up duration was 5.8 ± 3.30 years. Symptoms were recorded for four patients and included nyctalopia, constriction of visual fields, and blurred central vision. Mean BCVA were 1.92 ± 1.23 logMAR OD and 1.82 ± 1.31 logMAR OS at baseline examination and 2.44 ± 1.11 logMAR OD and 2.54 ± 0.91 logMAR OS at follow up. Optos ultrawide-field pseudo colour imaging showed macular atrophy, pigmentary changes in the mid to far peripheral retina, vascular attenuation, and pale optic discs. FAF showed macular atrophy associated with atrophy in the mid-peripheral retina, and 5/6 patients had slightly raised AF surrounding the area of macular atrophy. OCT imaging showed extensive outer retinal loss in 5/6 patients (Figure 4A–E). Patient 13 was the only patient with symptom onset after the age of 20. This patient had bilateral foveal sparing disease observed as an island of raised AF within the atrophic macular on FAF, and a region of relatively preserved ONL architecture surrounded by outer retinal atrophy on OCT imaging (Figure 4F).

### 3.2. Genetic Analysis

**Figure 5:**
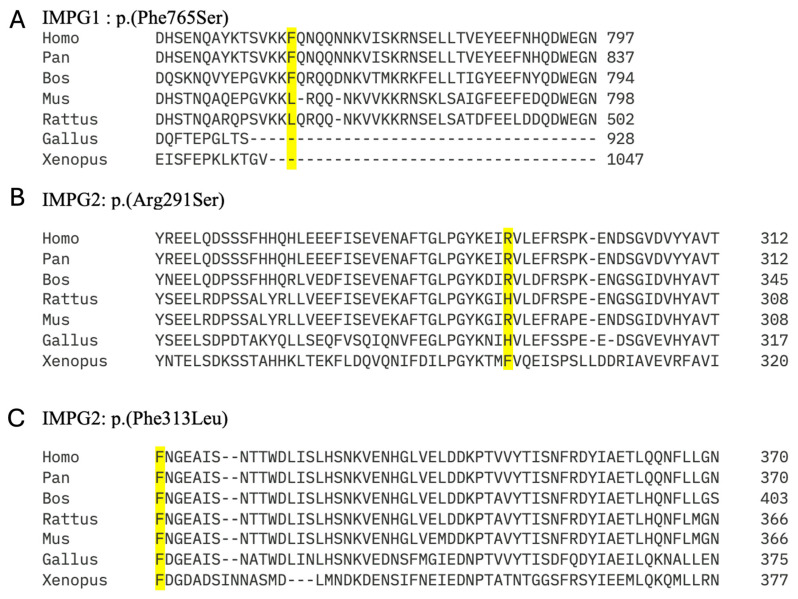
Analysis of evolutionary conservation in novel missense variants in *IMPG1* and *IMPG2* detected in this study.

Two different variants were detected in *IMPG1*; a novel missense c.2294T>C p.(Phe765Ser) variant and a deletion of exons 13 and 14 (Table 3). However, the c.2294T>C variant was assigned as a variant of unknown significance (VUS) as it was only predicted to be pathogenic by SIFT and was only conserved across 2 out of 7 different species (Figure 5A). Both of these patients had a phenotype consistent with PD.

**Figure 6:**
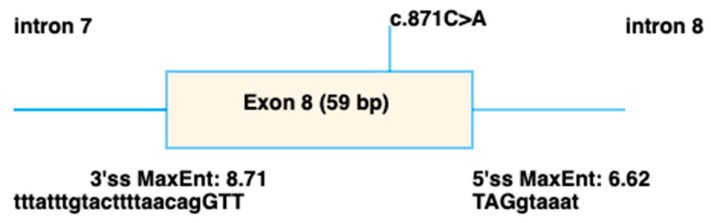
Schematic showing the splicing defect predicted to be caused by the c.871C>A p.(Arg291Ser) variant in *IMPG2*, using spliceAI visual [34].

The 11 variants detected in *IMPG2* included 4 missense and 7 truncating variants. Of the five novel *IMPG2* variants detected, three were frameshift variants predicted to result in truncated proteins and likely to result in loss of function due to nonsense mediated decay (NMD). The novel c.937T>C p.(Phe313Leu) was conserved across species (Figure 5C) and predicted to be pathogenic by all three in silico analysis programmes. By contrast, the novel c.871C>A p.(Arg291Ser) was only conserved in 4/7 species (Figure 5B) and was classified as benign by all three in silico analysis programmes. However, SpliceAI predicted it to cause an acceptor loss at 42bp upstream (intron7-exon8 boundary), and a donor loss at 16bp downstream (exon8-intron 8 boundary) (Figure 6).

### 3.3. Genotype–Phenotype Correlation

At least one truncating variant was identified in 10/11 *IMPG2* patients. The seven truncating variants are expected to produce a null protein due to nonsense mediated decay. Patient 5 who had AVMD was the only patient heterozygous for a missense variant (c.937T>C p.(Phe313Leu)). Of the six patients with RP, three patients were homozygous for truncating variants, and three patients carried a truncating and missense variant. The missense variants detected in patients with an RP phenotype included c.3193G>A p.(Gly1065Arg), c.871C>A p.(Arg291Ser), and c.391C>T p.(Arg131Cys). Of the four missense variants detected in our cohort, two were in the SEA1 domain, one was in the EGF2 domain, and c.391C>T p.(Arg131Cys) was located near the SEA1 domain.

Patient 13 who was compound heterozygous for c.963delC p.(Thr322fs) and c.391C>T p.(Arg131Cys) had the latest onset of disease and also had relatively preserved BCVA consistent with a small, spared area island of foveal tissue observed on FAF and OCT imaging. The remaining two RP patients who carried missense variants did not appear phenotypically different from the patients carrying truncating variants.

## 4. Discussion

Herein we report the phenotypic spectrum and genotypes of thirteen patients with either *IMPG1* or *IMPG2* retinopathy. *IMPG1* and *IMPG2* are essential for interphotoreceptor matrix function and photoreceptor maintenance. In our cohort, we demonstrate three distinct phenotypes as follows: pattern dystrophy in both the monoallelic *IMPG1* cases, maculopathy (PD/AVMD) in the monoallelic *IMPG2* cases, and retinitis pigmentosa with early maculopathy in biallelic *IMPG2* cases. These phenotypic findings align with those previously reported in the literature [5,6,7,8,27].

The phenotype of monoallelic *IMPG1* and *IMPG2* patients was characterised by late symptom onset with only one patient reporting symptoms before the age of 45 years. BCVA was also relatively preserved at first presentation and only one patient had BCVA ≤0.30 logMAR, consistent with previous findings by Brandl et al. [8]. However, long-term follow up in two of our patients showed a deterioration of vision reaching 0.90 logMAR, thus highlighting the progressive nature of the condition. This deterioration in BCVA supports the proposed natural history of *IMPG2* maculopathy starting as hyper-reflective material within the vitelliform lesion which can be resorbed leading to an optically empty cavity with relatively well-preserved RPE/Bruch’s membrane and this is followed by central macular atrophy with RPE loss [8]. An empty cavity was only observed in two of our patients and progression towards central macular atrophy was only observed in patient 4’s left eye; however, the OCT imaging two years prior to the atrophy in this eye showed a vitelliform lesion containing hyper-reflective material, so we cannot confirm the occurrence of the optical cavity in this eye. The morphological characteristics of the optical gap are clearly distinct from subretinal fluid related to choroidal neovascularisation or central serous chorioretinopathy (CSC) as it lacks features such as a pigment epithelial detachment, or gravitational tract on autofluorescence.

Drusen-like material was only observed in one monoallelic *IMPG1* patient. Of note, Meunier et al., reported this feature in 9/11 of their *IMPG1* retinopathy patients but none of their patients with *IMPG2* retinopathy [6]. Birtel et al., reported drusen in only one patient, pseudo-reticular drusen in their cohort of monoallelic *IMPG2* patients [7]. One of our monoallelic *IMPG2* patients had unilateral AVMD (which was shallower and associated with hyper-reflective dots when compared to the remaining three patients). To our knowledge, unilateral AVMD has only been described in one other *IMPG2* patient; however, hyper-reflective dots were not a feature in this case [35]. EDT testing was not performed in our study but other studies have shown normal EOG [6,24,28,36] but the mfERG amplitudes can be reduced in the central rings [6] in *IMPG1* and *IMPG2* retinopathy. This finding could potentially help differentiate AVMD caused by *IMPG1* and *IMPG2* retinopathy from BEST1 disease which usually has an abnormal EOG with when normal retinal function can be present [37].

The choroid appeared thickened in four of our monoallelic patients and this was most pronounced in the three patients with vitelliform lesions. To our knowledge, this feature has not been previously described in *IMPG1* or *IMPG2* retinopathy. Pachychoroid is typically seen in CSC where it is associated with the accumulation of subretinal fluid [38], which the empty adult vitelliform lesion in *IMPG2* retinopathy can be mistaken for. In CSC, Fluorescein angiography (FA) can help identify active fluid leakage and indocyanine green angiography (ICGA) can delineate choroidal abnormalities [38,39]. Our patients did not undergo FA, ICGA, or enhanced depth imaging (EDI) OCT, which limits our ability to draw clear conclusions about the relationship between pachychoroid and the adult vitelliform/PD phenotype in *IMPG1* and *IMPG2* retinopathy. [39].

In our biallelic *IMPG2* patients with RP, the mean age of onset was 18.4 years, which is later than 10.5 years reported by Van Huet et al. [5]. All our patients had macular atrophy, consistent with previous reports [5,27]. The severe reduction in BCVA in our RP patients was consistent with Van Huet et al.’s finding that BCVA were lower than 1.18 logMAR in all but one of their patients [5]. Of note, one patient had bilateral foveal sparing disease with relatively preserved BCVA (0.3 and 0.2 logMAR) at age 71 which to our knowledge has only been reported in two other patients in the literature. The patient reported by Van Huet et al. had BCVA of 0.18 logMAR aged 59 and the patient reported by Yuan et al. had BCVA of 0.54 logMAR OD and 0.18 logMAR OS aged 83 [5,25], potentially signifying relative preservation of BCVA in the later stages of disease, thus quantifying maintenance of visual function in patients with foveal sparing disease in RP secondary to pathogenic variants in *IMPG2*. Interestingly, both our patient and Yuan et al.’s patient with foveal sparing disease had symptom onset in their 40s and also carried the c.391C>T p.(Arg131Cys) variant. Our patient also carried the truncating c.963delC p.(Thr322fs) variant whilst Yuan et al.’s patient carried the c.3056G>A p.(Cys1019Tyr) in the EGF-like domain which the authors proposed caused significant changes to the interaction network, thus affecting the stability of the EGF-like domain and affecting the protein’s stability [25]. EDT was not performed in our cohort, but Van Huet et al. previously reported unrecordable ERG in 69% and severely reduced rod and cone function in the remaining 31% of patients [5].

Analysis of the genetic results in our cohort provides insights into genotype–phenotype correlation and disease mechanisms. Truncating variants were predominant in our *IMPG2* patients, with only one patient with maculopathy carrying a missense variant. This supports the hypothesis that haploinsufficiency may be the underlying mechanism in *IMPG2* retinopathy due to the prevalence of truncating *IMPG2* variants in both RP and maculopathy phenotypes, and the absence of these variants in control populations [7,8]. Interpretation of the effect of missense variants can be difficult, for example, spliceAI predicted a splicing defect for p.(Arg291Ser) variant in *IMPG2* despite the remaining in silico analysis programmes classifying it as benign. The novel p.(Phe313Leu) variant was the only missense variant in our monoallelic patients with maculopathy and this was predicted to be pathogenic by all three in silico analysis programmes but we are unable to comment on the possibility of this variant having a null-like effect secondary to misfolding as it caused AVMD in a heterozygous state despite not being expected to produce a truncated protein that eventually undergoes nonsense mediated decay. Indeed, missense variants in *IMPG2* have been shown to have variable effects. For example, c.370C>T p.(Phe124Leu) has been reported in a 63-year-old homozygous patient with mild maculopathy. Bandah-Rozenfeld et al. proposed that this missense variant may result in a partially functional protein, thus contributing to the milder observed phenotype in their homozygous patient. They suggested that missense variants in *IMPG2* may be associated with milder phenotypes compared to nonsense variants. However, Birtel et al. found that five missense variants identified in their monoallelic patients were predicted to have a significant reduction in function and protein misfolding, thus supporting the haploinsufficiency hypothesis and that some missense variants have an equivalent effect to loss of function variants [7]. Our data support Birtel et al.’s proposal of haploinsufficiency as a potential mechanism in *IMPG2* retinopathy as 4/5 of our *IMPG2* maculopathy patients were heterozygous for a truncating variant and all six patients with RP had at least one truncating variant, of which three had two truncating variants. However, Birtel et al. also noted incomplete penetrance in patients carrying truncating variants which led the authors to suggest that symptomatic disease cannot be predicted based on haploinsufficiency and that variable expressivity could be due to unknown genetic and environmental modifiers. Indeed, the missense variant identified in Bandah-Rozenfeld might only result in enough reduction in function of IMPG2 to cause disease when it is in the homozygous state. These findings could mean that *IMPG2* could be a suitable candidate gene for AAV gene therapy because can be packaged into an adeno-associated virus vector and it also appears to follow a loss of function mechanism.

Of note, the patient with *IMPG1* in our cohort with the c.2294T>C p.(Phe765Ser) variant had a PD phenotype different from the sectoral RP phenotype reported in Yuan et al.’s publication regarding a patient with the nearby p.(Leu740Phe), located near the EGF like domain. This phenotypic difference could be a result of a yet unknown functional difference between these two variants. However, reporting variants involving the EGF adds to the literature as variants are more commonly located in the SEA domains of *IMPG1* [25].

Animal models of *Impg1* and *Impg2* have provided important insights into the pathogenic mechanisms and the associated phenotypes. Salido et al. found that in *Impg1* knock-out mice did not show structural or functional deficits [14]. By contrast, Olivier et al. found that their *Impg1* knock-out mice had an abnormal accumulation of multi-focal subretinal deposits, photoreceptor degeneration along with a reduction in the length of the OS in rods and cones, and disruption of the IPM and RPE [40]. Olivier et al. suggested that the differences compared to the Salido study stemmed from their assessment of older mice since they propose that retinal degeneration in *Impg1* knock-out mice is only detectable after 9 months of age [40] (whereas those in the Salido et al. study were younger than 8 months).

In the *Impg2* knock-out mouse model, Impg1 abnormally accumulated within the subretinal space, significantly contributing to the subretinal lesions and the accompanying microglial activation. These mice also had reduced photopic and scotopic ERG responses [14,41]. Interestingly, Salido et al. found that knocking-out both *Impg1* and *Impg2* was associated with normal retinal structure (on SD-OCT) and function (scotopic and photopic ERG). This led the authors to propose that the visual deterioration associated with *Impg2* in mice was related to the incorrect distribution of the Impg1 protein [14].

By contrast, Xu et al. found that *Impg2* knock-out mice developed an RP phenotype with extinguished ERG and progressive photoreceptor degeneration. Rod photoreceptors were disorganised with rhodopsin mislocalised to the inner segments, whilst cone photoreceptors showed progressive loss with abnormal cell elongation [41]. The authors proposed that the misarrangement of the photoreceptors was due to the absence of Impg2 as a scaffold protein within the ECM. Moreover, there was an increase in sequestosome 1 (SQSTM1) and proteins that was suggestive of autophagy [41].

The phenotypic differences between the *Impg2* knockout models likely reflect the distinct CRISPR target used. Xu et al. generated two exon 2 mutations, 1) a 166 bp frameshift deletion that generated a truncated Impg2 protein that lacked the majority of the C-terminal domains and 2) three small exon 2 deletions (c.107_123del, c.269_274del, and c.276delG). By contrast, Salido et al. generated a 7 bp deletion in exon 3 that introduced a premature stop; Impg2 was undetectable using immunostaining, consistent with a null variant.

Williams et al., further investigated homozygous knock-in mice carrying the Q244*, T907*, and Y250C variants that had previously been identified in humans [42]. Q244* was chosen because it was thought to abolish Impg2 expression. Mice homozygous for Q244* and T907* had severe retinal disease characterised by the rapid development of gliosis, reduced photoreceptor outer segment preservation, outer retinal layer disruption, neurosensory detachment, and development of subretinal deposits adjacent to the optic disc and white retinal deposits that had features consistent with the AVMD phenotype in humans. By contrast, homozygous Y250C mice showed minimal late-onset retinal changes which included small drusen-like deposits thus suggesting a milder phenotype [42].

Additionally, Mayrel et al. found that the outer segment photoreceptor layer was absent in retinal organoids that were modelled on the *IMPG2*-RP phenotype. This led the authors to propose that the IPM is destabilised in the absence of IMPG2, thus making the outer segments susceptible to physical stress. Supporting this, the outer segments re-formed when the organoids were transplanted into the subretinal space of mouse eyes [43].

Our study has several limitations. The relatively small cohort size, with only two patients with *IMPG1*, limits our ability to draw definitive conclusions regarding genotype-phenotype correlations. The retrospective nature of the study meant that not all data were available for all patients and there were limited follow-up data to fully characterise disease progression. The relevance of pachychoroid in a limited number of our monoallelic patients could not be investigated further as we did not have EDI-OCT, FA, or ICGA imaging. Familial segregation studies were only available in one family, and family surveys were not performed; thus, possible asymptomatic carriers may be missed. Finally, the pathogenicity of detected variants was only investigated using in silico analysis. These limitations are frequently encountered in genotype–phenotype correlation studies. Future studies with longer follow up with familial surveys and segregation studies are required to accurately characterise the natural history of *IMPG1* and *IMPG2* retinopathies and also identify penetrance in monoallelic individuals. We were unable to perform functional analysis on the detected variants and future studies investigating the functional effects of the variants and especially novel missense variants will be helpful towards providing further evidence for their biological effect and their pathogenicity.

## 5. Conclusions

In conclusion, our findings show distinct phenotypic patterns of PD and AVMD in monoallelic *IMPG1* and *IMPG2* patients and RP with maculopathy in biallelic *IMPG2* patients. The predominance of truncating *IMPG2* variants supports haploinsufficiency as the underlying mechanism in maculopathy phenotypes. These insights improve our ability to diagnose *IMPG1* and *IMPG2* retinopathy and have important implications for genetic testing, clinical management, and potential future therapeutic strategies.

## Figures and Tables

**Table 1:** Genetic characteristics of patients with *IMPG1* and *IMPG2* retinopathy.

Patient	Variant 1	Protein Effect	Variant 2	Protein Effect	Other Variants	Phenotype
***IMPG1***						
P1	c.2294T>C	p.(Phe765Ser)	-	-	-	PD
P2	exon 13 and 14 deletion	-	-	-	-	PD
***IMPG2***						
P3	c.3229dupT	p.(Cys1077Leufs*2)	-	-	-	PD
P4	c.3162T>A	p.(Cys1054*)	-	-	-	AVMD
P5	937T>C	p.(Phe313Leu)	-	-	*FSCN2* c.49G>A p.(Val17IIe)	AVMD
P6	c.3023-6_3030dup	p.(Ala1011Phefs*2)	-	-	-	AVMD
P7	c.3229dup	p.(Cys1077Leufs*2)	-	-	-	AVMD
P8	c.2730delG	p.(Met910fs)	c.2730delG	p.(Met910fs)	-	RP
P9	c.3023-6_3030dup	p.(Ala1011Phefs*2)	c.3023-6_3030dup	p.(Ala1011fs)	*GRM6* c.19G>A p.(Ala7Thr) *PDE6C* c.1379C>T p.(Thr460Iie) *RP1* c.5624G>C p.(Gly1875Ala)	RP
P10	c.3413_3420delinsAATA	p.(Ser1138fs)	C.3193G>A	p.(Gly1065Arg)	*ABCA4* c.1928T>G p.(Val643Gly)	RP
P11	c.411G>A	p.(Trp137*)	c.871C>A	p.(Arg291Ser)	-	RP
P12	c.3023-6_3030dup	p.(Ala1011Phefs*2)	c.3023-6_3030dup	p.(Ala1011Phefs*2)	-	RP
P13	c.963delC	p.(Thr322fs)	c.391C>T	p.(Arg131Cys)	-	RP

**Table 2:** Clinical characteristics of patients with *IMPG1* and *IMPG2* retinopathy. AD, autosomal dominant, AVMD, adult vitelliform macular dystrophy, BCVA, best corrected visual acuity, F, female, HM, hand movements, M, male, N/A, NR, not recorded, NPL, no perception of light, PD, pattern dystrophy, POL, perception of light, RP, retinitis pigmentosa.

Patient Name	Sex	Allelic Status	Age of Onset	Symptoms	Family History	Baseline BCVA		Age	BCVA at Last Follow Up		Age	Phenotype
P1	F	Monoallelic	NR	NR	NR	0.42	0.52	81	N/A	N/A	N/A	PD/AVMD
P2	M	Monoallelic	NR	Asymptomatic	Sporadic	−0.10	−0.10	72	0.18	0.18	86	PD
P3	F	Monoallelic	40	Asymptomatic	Sporadic	0.00	0.00	40	0.00	0.00	55	PD
P4	M	Monoallelic	61	Central scotoma, delayed dark adaptation, photosensitivity	Sporadic	0.30	0.00	61	0.90	0.60	80	AVMD
P5	F	Monoallelic	69	Asymptomatic → blurred vision	AD (father had poor central vision)	0.26	−0.14	70	0.40	0.80	78	AVMD
P6	M	Monoallelic	56	Asymptomatic	N/A	0.10	0.18	57	N/A	N/A	N/A	AVMD
P7	M	Biallelic	45	Asymptomatic	Sporadic	0.10	−0.10	46	N/A	N/A	N/A	AVMD
P8	F	Biallelic	NR	Not recorded	Brother	POL	HM	78	N/A	N/A	N/A	RP
P9	M	Biallelic	4	Not recorded	Paternal grandmother	HM	HM	69	POL	HM	75	RP
P10	F	Biallelic	10	Nyctalopia, blurred central vision	N/A	1.48	0.95	48	POL	1.18	56	RP
P11	M	Biallelic	13	Constricted peripheral fields and nyctalopia	Sporadic	0.78	0.78	63	0.78	HM	71	RP
P12	M	Biallelic	20	Nyctalopia, constricted visual fields, and blurred central vision (later)	AR; sister, and nephew	NPL	NPL	83	NPL	NPL	84	RP
P13	M	Biallelic	45	Photosensitivity, photopsia, blurred central vision, and nyctalopia	Early onset visual loss paternal uncle	0.30	0.20	71	N/A	N/A	N/A	RP

**Table 3:** Summary of in silico analysis results for detected *IMPG1* and *IMPG2* variants.

Variant	Amino Acid	Mutation Type	GnomAD	Mutation Taster	Polyphen-2	SIFT	Alphamisse Class	CADD Score	SpliceAI Score	Novel	ACMG Class
***IMPG1***											
c.2294T>C	p.(Phe765Ser)	Missense	NR	Polymorphism	Benign	Not tolerated	Likely benign	16.6	0.01	Y	3
exon 13 and 14 deletion		SV	-	-	**-**	-	-	-	-		5
***IMPG2***											
c.391C>T	p.(Arg131Cys)	Missense	3.89 × 10^−5^	Disease causing	Possibly damaging	Not tolerated	Likely benign	25.4	0.10	N	3
c.411G>A	p.(Trp137*)	Nonsense	1.19 × 10^−5^	Disease causing	-	-		39	0.16	N	5
c.871C>A	p.(Arg291Ser)	Missense	NR	Polymorphism	Benign	Tolerated	Likely benign	19.8	0.64	Y	3
c.937T>C	p.(Phe313Leu)	Missense	NR	Disease causing	Probably damaging	Not tolerated	Likely pathogenic	22.4	0.00	Y	3
c.963delC	p.(Thr322fs)	Frameshift	NR	Disease causing	-	-	-		0.00	Y	5
c.2730delG	p.(Met910fs)	Frameshift	3.99 × 10^−6^	Disease causing	-	-	-		0.23	Y	5
c.3023-6_3030dup	p.(Ala1011Phefs*2)	Frameshift	1.78 × 10^−5^	-	-	-	-		0.95	N	5
c.3162T>A	p.(Cys1054*)	Nonsense	NR	Disease causing	-	-	-	36	0.00	Y	5
c.3193G>A	p.(Gly1065Arg)	Missense	3.98 × 10^−6^	Disease causing	Probably damaging	Not tolerated	Likely pathogenic	32	0.09	N	3
c.3229dupT	p.(Cys1077Leufs*2)	Frameshift	1.06 × 10^−5^	Disease causing	-	-	-		0.01	N	5
c.3413_3420delinsAATA	p.(Ser1138fs)	Frameshift	NR	Disease causing	-	-	-		0.03	N	5

## Data Availability

Dataset available on request from the authors.

## Supplementary Materials

The following supporting information can be downloaded at https://www.mdpi.com/article/10.3390/genes16121474/s1, Table S1: Lens status and other co-morbidities

## Abbreviations

The following abbreviations are used in this manuscript:

AVMD	Adult vitelliform macular dystrophy
AF	Autofluorescence
BCVA	Best corrected visual acuities
EZ	Ellipsoid zone
FAF	Fundus autofluorescence
IMPG	Interphotoreceptor matrix proteoglycan
IPM	Interphotoreceptor matrix
IRD	Inherited retinal disease
OCT	Optical coherence tomography
NGS	Next-generation sequencing
OD	Right eye
OS	Left eye
PD	Pattern dystrophy
RP	Retinitis pigmentosa
RPE	Retinal pigment epithelium
SPACR	Sialoprotein associated with cones and rods
SPACRCAN	Sialoprotein associated with cones and rods proteoglycans
SQSTM1	Sequestosome 1
VUS	Variant of unknown significance
