# Supplementary material for: Expanding the Genetic Spectrum in IMPG1 and IMPG2 Retinopathy
Source: Genes (Basel). 2025 Dec 9;16(12):1474. doi: 10.3390/genes16121474 (PMC12732860; doi:10.3390/genes16121474)
Supplement: Supplementary file 1 [file genes-16-01474-s001.zip › genes-3989184-supplementary.pdf]

Supplementary Table S1 Lens status and other co-morbidities.

Patient	Lens status	Other co-morbidities
Patient 1	Early cataract (non significant)	On tamoxifen
Patient 2	Cataract surgery aged 85 (PCIOL OD and antepupillary lens OS)	Post capsular tear with anterior vitrectomy OS<br>Hypertension<br>Hearing difficulties
Patient 3	Early cataract (non significant)	Smoker
Patient 4	Pseudophakia OU	
Patient 5	Pseudophakia OU	Hypermetropic
Patient 6	Early cataract (non significant)	Myopia
Patient 7	Early cataract (non significant)	
Patient 8	Not available	
Patient 9	Pseudophakia OU	
Patient 10	Pseudophakia OU	
Patient 11	Pseudophakia OU	Hypertension
Patient 12	Pseudophakia OU	
Patient 13	Early cataract (non significant)	
